# Biomarker alterations associated with distinct patterns of metastatic spread in colorectal cancer

**DOI:** 10.1007/s00428-020-02983-6

**Published:** 2020-12-09

**Authors:** M. Michl, F. Taverna, J. Kumbrink, T. S. Schiergens, V. Heinemann, J. Engel, T. Kirchner, Jens Neumann

**Affiliations:** 1 Department of Medicine III, University Hospital, LMU Munich, München, Germany; 2 Comprehensive Cancer Center, University Hospital, LMU Munich, München, Germany; 3 Institute of Pathology, Faculty of Medicine, LMU Munich, Thalkirchner Str. 36, D-81377 München, Germany; 4 German Cancer Consortium (DKTK), German Cancer Research Centre (DKFZ), Heidelberg, Germany; 5 Department of General, Visceral and Transplantation Surgery, University Hospital, LMU Munich, München, Germany; 6 Munich Cancer Registry (MCR), Ludwig-Maximilians-University of Munich, Munich, Germany; 7 Institute of Medical Informatics, Biometry and Epidemiology (IBE), Ludwig-Maximilians-University of Munich, München, Germany

**Keywords:** Lung metastasis, Metastatic colorectal cancer, Biomarker, CD133, β-catenin, MAPK pathway mutations

## Abstract

Metastatic spread is the most important life-threatening feature of colorectal cancer and is supposed to be mainly driven by alterations in different carcinogenic pathways. The present study compared mutation and expression profiles of distinctive biomarkers in colorectal cancer patients with different clinical metastatic patterns. As for a case-control study, patients were matched according to T category, grading and primary tumour site. Overall, 246 patients with either exclusive lung metastasis (*N* = 82), exclusive liver metastasis (*N* = 82) or non-metastatic colorectal cancer (*N* = 82) were identified. Paraffin-embedded specimens were examined for mutations in the *RAS* and *RAF* genes and for the expression of β-catenin and CD133. Clinical endpoints were presence or absence of distant metastasis, formation of metastasis in lungs versus the liver and survival. MAPK pathway mutations in either the *KRAS*, *NRAS* or *BRAF* gene were associated with the development of lung metastasis (63.4%) compared to the control group (47.6%; *p* = 0.04). MAPK pathway alterations plus high β-catenin expression were associated with metastasis to the lungs but not to the liver (28.0% vs. 13.4%; *p* = 0.02). High CD133 expression correlated with the development of liver metastasis compared to the control group (30.5% vs. 14.6%; *p* = 0.02). This data indicates that different patterns of distant spread are associated with specific biomarker alterations and may represent different molecular subtypes of colorectal cancer. However, underlying mechanisms of metastasis formation in different anatomic sites remains unclear. Since knowledge of the anticipated site of distant spread would substantially impact clinical management, further research is needed to identify solid biomarkers for different metastatic patterns.

**Supplementary Information:**

The online version contains supplementary material available at 10.1007/s00428-020-02983-6.

## Introduction

Organ metastasis is still the leading cause of death in the majority of colorectal cancer (CRC) patients. Prognosis greatly differs inter-individually and crucially depends—inter-alia—on metastatic patterns [1]. Organs most commonly affected are the liver (50–70%) and lungs (10–30%) [2]. Today, management of metastatic CRC (mCRC) includes multidisciplinary approaches combining surgery, systemic therapy and local ablative techniques in order to provide personalized treatment procedures [3–6]. Thus, a reasonable effort was put into the identification of prognostic markers for predicting the clinical course and individual prognosis of patients with mCRC [7–9]. The *RAS* and *BRAF* mutational status as well as the mismatch repair (MMR) status represent acknowledged biomarkers in mCRC patients as they have a prognostic impact and influence systemic treatment [10–12]. Besides its prognostic and predictive relevance, the *RAS* mutational status was shown to impact the metastatic phenotype in mCRC [13]. Whilst the presence of a *RAS* mutation seems to constitute an independent risk factor for the development of lung, brain and bone metastasis, *RAS* wild-type tumours are associated with the presence of liver metastasis [14–18].

However, apart from these analyses, no established biomarkers exist for predicting the formation of metastasis in the lungs or the liver to date. In previous studies, our group demonstrated that the expression of CD133 and, in particular, the combined expression of CD133 and β-catenin, both associated with the Wnt-/β-catenin-pathway and stem cell features of tumour cells, significantly correlates with poor prognosis as well as the formation of distant metastasis in the liver [19–21]. Further analyses revealed that the expression of these markers did not correlate with the presence of peritoneal carcinomatosis or brain metastasis [22, 23]. We hypothesized that different mechanisms must play a role in the development of cavitary metastasis, brain metastasis and metastases in the liver. On that basis, the question arose whether both markers could also play a crucial role in the development of pulmonary metastasis.

Within the present study, we examined CRC specimens from patients with exclusive lung metastasis (PUL group) and exclusive liver metastasis (HEP group) and compared them with tumours deriving from patients without distant metastases that served as the control group (M0 group). Based on the data from the literature and the results from our study group, we focused on the investigation of single biomarkers consisting of MAP kinase pathway mutations as well as β-catenin and CD133 expression status and analyzed their impact on the formation of lung or liver metastasis and survival. Furthermore, the association of different biomarker combinations with different localizations of distant metastases was evaluated.

As most data in the literature is deducted from retrospective-exploratory analyses or epidemiological studies, we specifically chose a case-control format as study design in order to eliminate competing for confounding factors by matching all study patients according to the most relevant clinical and pathological criteria in CRC.

## Material and methods

### Patients

All patients involved in the present analysis were diagnosed at the Institute of Pathology, Faculty of Medicine, LMU Munich, and subsequently identified via systematic database search in collaboration with the Munich Cancer Registry (MCR). The MCR covers an estimated population of meanwhile 4.9 million inhabitants in the southern part of Germany. Search items comprised “colorectal cancer” *and* “exclusive lung metastasis” (for the creation of the PUL group), “colorectal cancer” *and* “exclusive liver metastasis” (for the generation of the HEP group) and “colorectal cancer” *and “*no organ metastasis” *and* “no local recurrence” within 5 years after first CRC diagnosis (for the formation of the M0 group). Patients with a histologically proven diagnosis of CRC and the histological or radiological diagnosis of lung or liver metastasis reported to the MCR between 1998 and 2017 were considered for the PUL or HEP group, respectively. As a control, the M0 group consisting of CRC patients with non-metastatic disease reported to the MCR between 1998 and 2012 was assembled. By halting the recruitment of patients for the M0 group in 2012, non-metastatic and recurrence-free survival during a follow-up period of at least 5 years was ensured. Available patient and tumour characteristics as well as survival data were collected. Patients with secondary malignancies were excluded.

### Study design

A matched-pair analysis was deemed appropriate for the present investigation. Patients from all groups were matched according to pT category, grading and primary tumour site. As suitable for a matched-pair analysis, all groups consisted of equal patient numbers. Availability of sufficient analyzable tumour tissue limited patient numbers to 82 patients per group (Online Resource 1).

### Immunohistochemistry

For immunohistochemistry, 5-μm whole-tissue sections of formalin-fixed and paraffin-embedded (FFPE) tumour samples were stained employing a Ventana Benchmark (Ventana Medical Systems, Oro Valley, AZ) following the manufacturer’s instructions. A detailed description of antibodies and protocols used in this study is provided in Online Resource 2. To exclude unspecific reactions of antibodies and/or reagents, isotype and system controls were performed.

### Scoring of immunohistochemistry

**Fig. 1:**
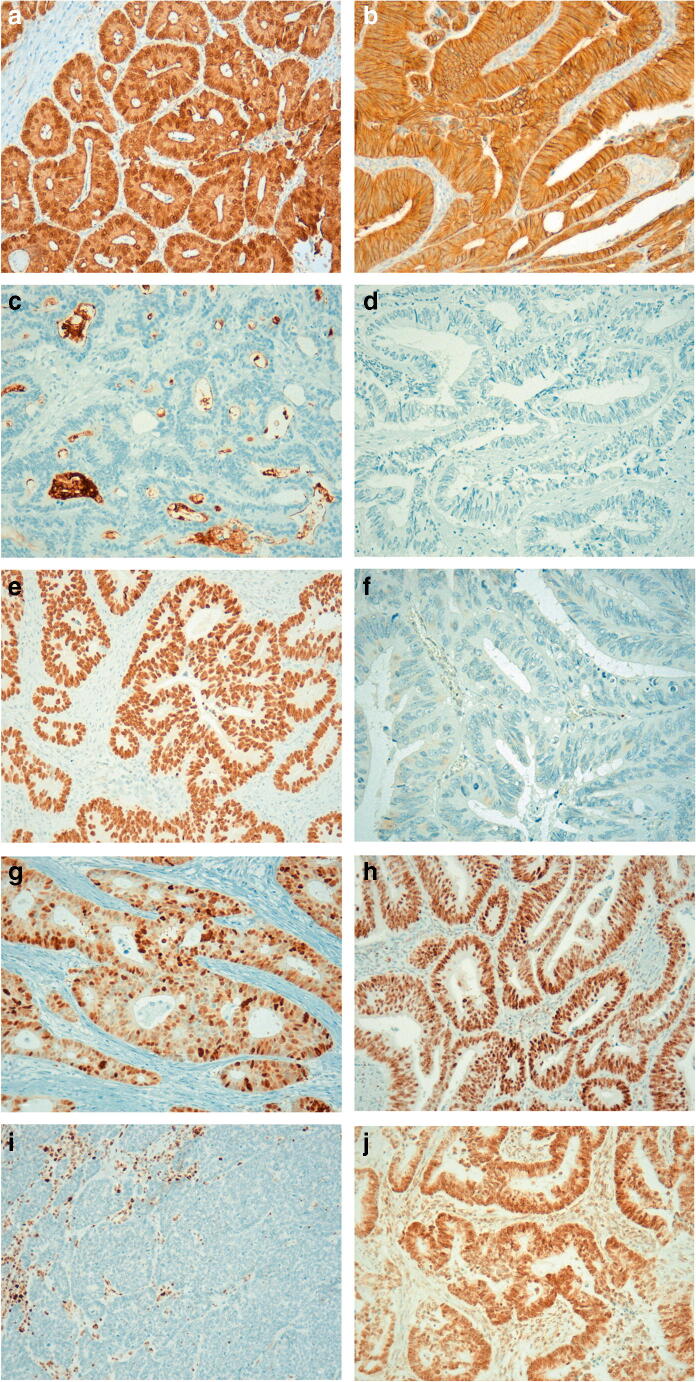
Immunohistochemical analysis in colorectal cancer specimens. **a** High nuclear β-catenin expression; **b** low nuclear β-catenin expression; **c** high CD133 expression; **d** low CD133 expression; **e** deregulated p53 expression pattern with diffuse strong nuclear p53 staining; **f** deregulated p53 expression pattern with the absence of nuclear p53 expression; **g** regulated p53 expression pattern; **h** positive MLH1-staining (pMMR); **i** negative MLH1-staining (dMMR); **j** positive MSH2-staining (pMMR)

All samples were evaluated independently by two investigators (J.N. and F.T.), both blinded for the clinical outcome. In case of discrepancy, samples were jointly reviewed and a consensus was reached. A staining score for nuclear expression of β-catenin was based on the quantity of stained tumour cell nuclei throughout the whole tumour, whereas the intensity of staining was not considered (Fig. 1a and b). The score was as follows: 0, negative; 1+, < 30%; 2+, 30–60%; 3+, > 60% positive cells. Subsequently, the cases were classified into low (scores 0 and 1) and high-grade (scores 2 and 3) expression. For CD133, protein expression was defined as either staining of apical membranous parts of the cells or of shed cellular debris in the tumour glands. CD133 expression levels were scored as low grade (< 50% of positive tumour glands, Fig. 1c) or high grade (≥ 50% positive tumour glands, Fig. 1d). Aberrant expression of p53 was defined as strong diffuse nuclear staining in > 90% of tumour cells (Fig. 1e) or complete absence of p53 expression in all tumour cells (Fig. 1f). A regulated p53 expression pattern is shown in Fig. 1g. Loss of MLH1 and MSH2 expression (reflecting a dMMR) was recorded when nuclear staining was absent in malignant cells but preserved in the stroma cells or normal epithelial cells, respectively. Cases with preserved nuclear MLH1 and MSH2 expression in tumour cells were classified as cases with proficient MMR status (Fig. 1h–j).

### Mutation analysis

For enrichment of tumour tissue, H&E-stained histological serial sections were inspected and areas containing tumour cells were defined and marked. These sections were used as blueprints for transferring the marked areas onto unstained dewaxed tissue sections. Then, marked areas were microdissected under microscopic control using scalpel blades. From the resulting tissue, DNA was isolated using QIAamp® DNA Micro Kit (Qiagen, Hilden, Germany) following the user’s handbook. For the detection of *BRAF*, *KRAS* and *NRAS* mutations, pyrosequencing was employed. Briefly, HotStarTaq® Polymerase (Qiagen, Hilden, Germany) was used together with 1 × PCR buffer (1.5 mM MgCl_2_), 200 μM dNTPs and 400-nM primers applying optimized PCR conditions (Online Resource 3). PCR products were analyzed using PyroMark® Gold Q24 kits (Qiagen, Hilden, Germany) together with 0.3 μM of the corresponding sequencing primer (Online Resource 3) employing the PyroMark® Q24 device (Qiagen, Hilden, Germany). Finally, data were analyzed applying the PyroMark® Q24 software (Qiagen, Hilden, Germany).

### Statistical analysis

For comparison of patient and tumour characteristics between groups, a two-sided *χ*^2^ test was used. Only “age” as a metric and not normally distributed variable was tested with the Kruskal–Wallis test (global *p*) and the Mann–Whitney *U* test (PUL vs. HEP). For global testing and head-to-head comparisons, the significance of correlations of single biomarkers and biomarker combinations was calculated using a *χ*^2^ test. Survival analysis for overall and progression-free survival was assessed using the Kaplan–Meier method and univariate survival distributions were compared using the log-rank test. Multivariate logistic regression analysis was performed in order to rule out confounding factors between groups. For multivariate analysis, the factors “age”, “lymph node status” (N0 vs. N+), “MAP kinase status”, “MMR status”, “p53 expression status”, “beta-catenin expression status” and “CD133 expression status” were considered. For all statistical tests, the SPSS V. 26.0 Software (IBM Inc., Armonk, NY) was employed. A *p* value lower than 5% (*p* < 0.05) was considered statistically significant.

## Results

### Patient and tumour characteristics

The entire study population consisted of 246 patients (145 male (59%), 101 female (41%)) with histologically proven adenocarcinoma of the colorectum and exclusive lung metastasis (PUL; *N* = 82), exclusive liver metastasis (HEP; *N* = 82) or non-metastatic disease (M0; *N* = 82). Baseline patient demographics and tumour characteristics of the analyzed patient cohort are presented in Table 1 and Online Resource 4. Patients differed between groups in a few non-matching variables such as gender, primary tumour site and pN category (global p). However, when comparing only patients with exclusive lung versus exclusive liver metastasis (PUL vs. HEP), baseline characteristics were equally distributed.

**Table 1:** Patient and tumour characteristics of the entire study population (*N* = 246)

Total *N* = 246	PUL *N* = 82	HEP *N* = 82	M0 *N* = 82	*P*	
				Global	PUL vs HEP
Sex					
Female	27 (32.9)	32 (39.0)	42 (51.2)	0.053	0.42
Male	55 (67.1)	50 (61.0)	40 (48.8)		
Age at first diagnosis of CRC					
Median, years	64.4	63.5	71.3	< 0.001	0.28
≥ 70 years	28 (34.1)	24 (29.3)	46 (56.1)	0.001	0.50
≥ 65 years	39 (47.6)	37 (45.1)	64 (78.0)	< 0.001	0.75
< 65 years	43 (52.4)	45 (54.9)	18 (22.0)		
Sidedness of primary					
Right colon	9 (11.0)	9 (11.0)	9 (11.0)	1.00	1.00
Left colon	73 (89.0)	73 (89.0)	73 (89.0)		
Primary tumour site					
Colon	25 (30.5)	32 (39.0)	51 (62.2)	< 0.001	0.29
Rectosigmoid	7 (8.5)	10 (12.2)	7 (8.5)		
Rectum	50 (61.0)	40 (48.8)	24 (29.3)		
Grading					
Low grade (G1, G2)	54 (65.9)	56 (68.3)	56 (68.3)	0.97	0.59
High grade (G3)	27 (32.9)	26 (31.7)	26 (31.7)		
Unknown	1 (1.2)	-	-		
pT stage					
pT0	2 (2.4)	-	-		
pT1	3 (3.7)	4 (4.9)	4 (4.9)		
pT2	11 (13.4)	11 (13.4)	8 (9.8)	0.50	0.49
pT3	57 (69.5)	58 (70.7)	60 (73.2)		
pT4	7 (8.5)	9 (11.0)	10 (12.2)		
Unknown	2 (2.4)	-	-		
pN status					
pN0	32 (39.0)	31 (37.8)	52 (63.4)		
pN1	31 (37.8)	37 (45.1)	26 (31.7)	0.001	0.14
pN2	14 (17.1)	14 (17.1)	3 (3.7)		
Unknown	5 (6.1)	-	1 (1.2)		
Time of metastasis					
Metachronous	42 (51.2)	23 (28.0)	NA	-	0.002
Synchronous	40 (48.8)	59 (72.0)	NA		

For each category, absolute patient numbers are given and percentages in brackets. *NA* not applicable

Median age at first CRC diagnosis was significantly higher in patients of the M0 group compared to the PUL and HEP groups (71.3 vs. 64.4 vs. 63.5 years; global *p* < 0.001). A higher proportion of patients from the M0 group presented with a primary tumour located in the colon (51 (62.2%)), whereas in the PUL and the HEP group, the majority of primaries was found in the rectum (50 (61.0%) and 40 (48.8%)) (global *p* < 0.001). Detailed subgroup analysis showed that more patients from the PUL group had their primary in the rectum compared to the M0 group (*p* < 0.001) and also more cases with rectal primaries were found in the HEP group compared to the M0 group (*p* = 0.01). No significant difference with regard to the primary tumour site was detected between the PUL and the HEP group (*p* = 0.29). Also, the temporal occurrence of metastasis was significantly different between groups. Whilst the majority of liver metastasis occurred synchronously (59 (72.0%)), lung metastasis was detected synchronously and metachronously in nearly equal parts (42 (51.2%) and 40 (48.8%); *p* = 0.002 for PUL vs. HEP).

### Frequency of single-biomarker alterations according to metastatic status

For each patient group, all detected MAP kinase (MAPK) pathway mutations and immunohistochemical expression profiles of CD133, β-catenin and p53 are listed in Table 2. Analyzed MAPK pathway alterations included the *RAS* mutational status of the *KRAS* and *NRAS* hotspots in exons 2, 3 and 4 as well as the *BRAF* V600 mutational status and other *BRAF* mutations. In decreasing order of frequency, MAPK pathway alterations were detected in the PUL (52 (63.4%)), HEP (42 (51.2%)) and M0 (39 (47.6%)) group (global *p* = 0.10). Thus, the highest frequency of MAPK pathway mutations was observed in patients with lung metastasis mainly consisting of *RAS* mutations (*N* = 48 (58.5%)) and hereunder especially of *KRAS* exon 2 mutations (*N* = 42 (51.2%)). Consistently, in the HEP and M0 group, *RAS* respectively *KRAS* exon 2 mutations represented the most common MAPK pathway mutations (HEP (41 RAS (50.0%) and 38 *KRAS* exon 2 mutations (46.3%)); M0 (35 RAS (42.7%) and 33 *KRAS* exon 2 mutations (40.2%)). One patient with liver metastasis was diagnosed with a double mutation in both the *KRAS* exon 3 and the *NRAS* exon 2, respectively. No difference in frequencies of *NRAS* and *BRAF* mutations was observed between groups (*p* = 0.60 and *p* = 0.25, respectively).

**Table 2:** Frequency of MAP kinase mutations and immunohistochemical expressions of CD133, beta-catenin and p53 comparing the three patient cohorts

	Total *N* = 246	PUL *N* = 82		HEP *N* = 82		M0 *N* = 82		Global *P*
MAP kinase mutational status								
MAP kinase mutation	133	52 (63.4)		42 (51.2)		39 (47.6)		0.10
MAP kinase wild type	113	30 (36.6)		40 (48.8)		43 (52.4)		
RAS mutation	124	48 (58.5)		41 (50.0)		35 (42.7)		0.13
RAS wild type	122	34 (41.5)		41 (50.0)		47 (57.3)		
KRAS mutation	117	46 (56.1)		38 (46.3)		33 (40.2)		0.12
Exon 2	103	42 (51.2)		33 (40.2)		28 (34.1)		
Exon 3	8	1 (1.2)		4 (4.9)^#^		3 (3.7)		
Exon 4	6	3 (3.7)		1 (1.2)		2 (2.4)		
KRAS wild type	129	36 (43.9)		44 (53.7)		49 (59.8)		
NRAS mutation	8	2 (2.4)		4 (4.9)		2 (2.4)		0.60
Exon 2	5	1 (1.2)		2 (2.4)^#^		2 (2.4)		
Exon 3	3	1 (1.2)		2 (2.4)		-		
Exon 4	-	-		-		-		
NRAS wild type	238	80 (97.6)		78 (95.1)		80 (97.6)		
BRAF mutation	9	4 (4.9)		1 (1.2)		4 (4.9)		0.35
V600E	8	4 (4.9)		1 (1.2)		3 (3.7)		
Other	1	-		-		1 (1.2)		
BRAF wild type	237	78 (95.1)		81 (98.8)		78 (95.1)		
Stem cell marker								
CD133 high	57	20 (24.4)		25 (30.5)		12 (14.6)		0.05
CD133 low	189	62 (75.6)		57 (69.5)		70 (85.4)		
Wnt pathway								
β-catenin high	109	41 (50.0)		33 (40.2)		35 (42.7)		0.43
β-catenin low	137	41 (50.0)		49 (59.8)		47 (57.3)		
p53 pathway								
p53 regulated	80	28 (34.2)		24 (29.3)		28 (34.2)		0.74
p53 deregulated	166	54 (65.8)		58 (70.7)		54 (65.8)		
MMR status								
pMMR	239	81 (98.8)		80 (97.6)		78 (95.1)		0.36
dMMR	7	1 (1.2)		2 (2.4)		4 (4.9)		
Marker combinations*		Positive	Negative	Positive	Negative	Positive	Negative	
MAP kinase mutational status PLUS β-catenin expression		23 (28.0)	59 (72.0)	11 (13.4)	71 (86.6)	14 (17.1)	68 (82.9)	*0.048*
MAP kinase mutational status PLUS CD133 expression		15 (18.3)	67 (81.7)	17 (20.7)	65 (79.3)	5 (6.1)	77 (93.9)	*0.02*
β-catenin expression PLUS CD133 expression		9 (11.0)	73 (89.0)	10 (12.2)	72 (87.8)	8 (9.8)	74 (90.2)	0.88

For each category, absolute patient numbers are given and percentages in brackets. Significant *p* values are printed in italics

*Positive is defined as both markers being positive (positive for MAP kinase mutational status is defined as the presence of a mutation; positive for β-catenin and CD133 expression is defined as a high expression). Negative is defined as one of the markers or both markers being negative (negative for MAP kinase mutational status is defined as wild type; negative for β-catenin and CD133 expression is defined as a low expression)

^#^One case with a double mutation in KRAS Exon 3 and NRAS Exon 2

Regarding the stem cell marker CD133, high immunohistochemical expression was most frequently detected in patients with liver metastasis (*N* = 25 (30.5%)), nearly reaching the level of significance in comparison with the PUL and M0 group (*p* = 0.05). Immunohistochemical detection of nuclear ß-catenin expression representing an active Wnt/β-catenin pathway did not reveal significant differences between groups as a single biomarker. The p53 status did not differ significantly between groups and was even considered almost identical. Seven out of 246 patients (2.9%) showed deficient mismatch repair status (dMMR). Most of them were found in the M0 group (*N* = 4/82 (4.9%)) followed by the HEP (*N* = 2/82 (2.4%)) and the PUL group (*N* = 1/82 (1.2%)). In the metastatic group (HEP and PUL), two out of three patients showed mutations in the *BRAF* gene, whereas no mutations in the analyzed *RAS* genes could be obtained. One out of two *BRAF*-mutated cases showed combined overexpression of CD133, the other case showed a coincidence of deregulated p53 status and deregulated CD133 status. In the M0 cohort with dMMR status, two *BRAF*-mutated cases were found, one of them with combined deregulated p53 status. One case showed exclusive *BRAF* mutation and one case exhibited combined β-catenin and CD133 expression. One patient showed no alterations in the analyzed pathways. Taken together, no distinct pattern of altered biomarkers or biomarker combinations could be obtained and due to low patient numbers, no level of statistical significance was obtained in the global analysis (*p* = 0.36) or head-to-head comparisons (Table 3).

**Table 3:** Head-to-head comparisons between groups according to single biomarkers and biomarker combinations

	M1 ↔ M0	PUL ↔ M0	HEP ↔ M0	PUL ↔ HEP
Single biomarker				
MAP kinase mutational status	0.15	*0.04*	0.64	0.11
CD133 expression	*0.03*	0.12	*0.02*	0.38
β-catenin expression	0.72	0.35	0.75	0.21
MMR status	0.18	0.17	0.41	0.56
Marker combinations				
MAP kinase mutational status PLUS β-catenin expression	0.50	0.09	0.52	*0.02*
MAP kinase mutational status PLUS CD133 expression	*0.01*	*0.02*	*0.01*	0.69
β-catenin expression PLUS CD133 expression	0.67	0.80	0.62	0.81

Significant *p* values are printed in italics

Multivariate analysis adjusting results for the most relevant confounders (age, lymph node status (N0 vs. N+), MAP kinase status, MMR status, p53, beta-catenin and CD133) confirmed the independent association between the investigated markers and metastatic patterns (Online Resource 5).

### Biomarker combinations associated with the formation of metastasis in the lungs or the liver

**Fig. 2:**
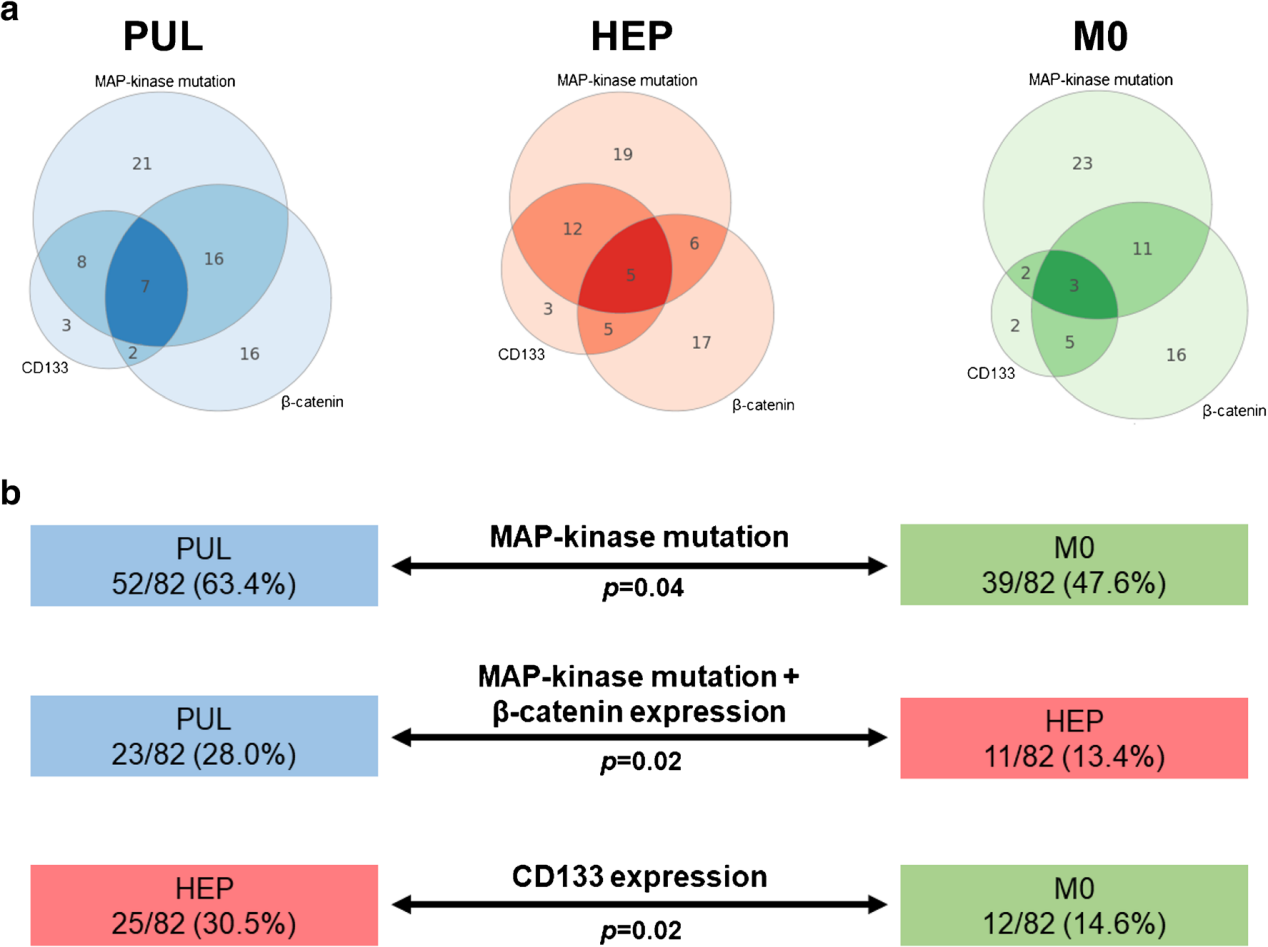
**a** Venn diagram illustrating the frequencies and coincidences of biomarker alterations according to metastatic status. Numbers indicate proven alterations which are defined as the presence of a mutation in the MAP kinase pathway and high immunohistochemical expression of Wnt/β-catenin and CD133 (see Table 2). **b** Overview of the relevant biomarker alterations correlating with the formation of metastasis in the lungs and/or the liver

When taking into account the abundance of biomarker combinations instead of single biomarkers, patients with exclusive lung metastasis (PUL group) were characterized by the presence of both a MAPK pathway mutation *plus* a high Wnt/β-catenin expression (*N* = 23 (28.0%)), whereas a larger proportion of patients from the HEP and M0 group showed negativity for one or both markers (global *p* = 0.048; PUL vs. HEP *p* = 0.02; Tables 2 and 3). Furthermore, MAPK pathway mutations in combination with high CD133 expression correlated with the development of metastasis in general compared to a non-metastatic course of the disease (M1 (PUL *N* = 15 (18.3%) and HEP *N* = 17 (20.7%)) vs. M0 *N* = 5 (6.1%); *p* = 0.01) (Table 3). Head-to-head comparisons of all groups according to single biomarkers and biomarker combinations as well as corresponding *p* values are summarized in Table 3. Frequencies and coincidence of alterations according to metastatic status are illustrated in Fig. 2a. Figure 2b gives an overview of the extracted results.

### Survival data according to biomarker profiles

According to biomarker profiles, survival analyses comparing overall survival (OS) and progression-free survival (PFS) of patients from the PUL versus the HEP group were performed. For OS analysis, 53 of 82 patients from the PUL group and 56 of 82 patients from the HEP group were available. For PFS, 77 of 82 patients from each the HEP and the PUL group were available.

Overall, patients with lung metastasis showed a longer OS and PFS than patients with liver metastasis (OS: 65.7 vs. 37.1 months, HR 1.41 [95% CI: 0.96–2.06], *p* = 0.08; PFS: 23.9 vs. 16.1 months, HR 1.45 [95% CI: 1.06–2.00], *p* = 0.02) (Online Resource 6 and Online Resource 7).

In both the PUL and the HEP group, the presence of a MAPK pathway mutation was associated with inferior OS (PUL: 51.5 vs. 83.6 months, HR 1.96 [95% CI: 1.07–3.60], *p* = 0.03; HEP: 26.4 vs. 46.3 months, HR 1.93 [95% CI: 1.13–3.29], *p* = 0.02) and PFS (PUL: 19.0 vs. 28.8 months, HR 1.99 [95% CI: 1.18–3.35], *p* = 0.01; HEP: 10.1 vs. 19.1 months, HR 1.53 [95% CI: 0.97–2.42], *p* = 0.07) compared to patients without MAPK pathway mutations. This effect stayed significant when comparing patients with and without MAPK pathway mutations between groups (OS: global *p* = 0.004, PFS: global *p* = 0.002) (Online Resource 6 and Online Resource 8).

Within the PUL group, the different marker combinations consisting of MAPK pathway mutations and CD133 expression status were associated with significant differences in OS and PFS. In patients with lung metastasis and a MAPK pathway mutation *plus* a high CD133 expression, both OS and PFS were significantly shorter than in patients with either MAP kinase wild-type tumours and/or low CD133 expression (OS: 48.1 vs. 67.4 months, HR 2.37 [95% CI: 1.20–4.67], *p* = 0.01); PFS: 19.0 vs. 28.5 months, HR 1.86 [95% CI: 1.01–3.43], *p* = 0.04) (Online Resource 6).

No survival differences were observed when focusing on CD133 and β-catenin expression as single biomarkers. Also, neither the combination of MAPK pathway mutations *plus* β-catenin expression status nor the combination of β-catenin *plus* CD133 expression status revealed any survival differences within or between groups.

## Discussion and conclusion

There is consensus that colorectal cancer is a heterogeneous disease. Also, it is widely accepted that tumour biology impacts treatment efficacy and long-term outcome [24–26]. However, when it comes to different metastatic phenotypes, no reliable biomarkers predicting the formation of distant metastasis exist so far. For clinical management, knowledge about the CRC patient’s individual risk to metastasize and the site of distant spread could guide aggressiveness of (local) treatment and help to stratify patients for personalized treatment approaches.

Thus, the present study aimed to investigate biomarker profiles that could predict the development of metastasis in the lungs or the liver, both pivotal organs and frequently affected by CRC metastasis. Indeed, we were able to identify biomarker profiles that are associated with different patterns of distant spread in colorectal cancer. We show that the presence of a MAPK pathway mutation *plus* a deregulation of the Wnt/β-catenin pathway is associated with metastasis to the lungs indicating that this biomarker combination could predict a higher risk of lung metastasis. In contrast, the expression of the stem cell-associated marker CD133 correlated with the presence of liver metastasis.

The clinical interest to estimate the likelihood of lung or liver metastasis or a non-metastatic course of disease clearly lies in follow-up care after local treatment of CRC and early detection of potentially resectable or locally treatable organ metastasis. Referring to an easy-to-practice biomarker profile that predicts the development of metastasis in the liver or the lungs could enable physicians to perform diagnostic procedures according to the patient’s individual risk and pave the way for early treatment of organ metastasis. With the MAPK pathway mutations, β-catenin as a representative from the Wnt/β-catenin-pathway as well as the stem cell marker CD133, we specifically examined standard biomarkers that are well established and widely used in routine diagnostics of CRC specimens.

To accomplish the highest statistical accuracy, we chose a matched-pair analysis and therefore created a biobank containing clinical data and tumour tissue specimen from 246 CRC patients with exclusive lung metastasis (PUL group), exclusive liver metastasis (HEP group) or non-metastatic disease (M0 group). Using this case-control design, patients were matched according to relevant baseline characteristics as appropriate for a matched-pair analysis to exclude confounding factors.

With regard to the biological background, CRC develops as a result of different combinations of genetic alterations, epigenetic changes and posttranslational modifications [27]. Most CRC arises through the gradual malignant progression of a benign precursor lesion known as an adenoma. The majority of sporadic CRC arises via the adenoma-carcinoma sequence starting with mutations of the tumour-suppressor adenomatous polyposis coli (APC) with subsequent dysregulation of the Wnt/β-catenin pathway [27]. Forced expression of mutant *KRAS* in CRC enhances nuclear β-catenin accumulation and increases the levels of Wnt target genes. This *KRAS*-induced augmentation of Wnt/β-catenin activity results in increased proliferation and malignant progression and therefore is an important driver for the formation of metastases [28, 29].

In our study, we demonstrate that a combined expression of β-catenin together with a MAPK pathway mutation correlates with metastasis to the lungs but not to the liver. Indeed, some clinical studies described MAP kinase mutations as independent risk factors for the development of lung metastasis [14–17]. We focused on the metastatic route into the lungs and compared them against the liver and non-metastatic disease and can clearly disclose a difference in the formation of metastasis to these organs. Based on the results of Horst and coworkers, who showed that the enhancement of the Wnt/β-catenin pathway leads to proliferation and malignant progression in vitro [29], we observe that this marker combination correlates with the formation of metastasis in the lungs but not in the liver.

Investigating the stem cell marker CD133, the subgroup of patients with liver metastasis showed the highest proportion of tumours with high CD133 expression compared to patients with lung metastasis or non-metastatic disease. In detail, 30.5% of patients with exclusive liver metastasis showed CD133 positivity and the percentage was comparable to previously reported results from Horst et al. that described 26% of unselected colorectal tumours as CD133 high and 74% as CD133 low [30].

Interestingly, the combination of a MAPK pathway mutation *plus* a high CD133 expression was associated with a distant spread in general (M0 vs. M1). This finding may be explained by the fact that the MAPK pathway mutation still depicts the dominant driver in formation of metastasis. Also, the combination of nuclear β-catenin expression *plus* CD133 expression did not reveal any significant association with metastatic patterns in the present analysis. This is not necessarily in contrast to previously published data from our group showing an association between the high expression of CD133 and/or nuclear β-catenin and a high risk of distant metastases in right-sided colon cancers [19]. As the majority (89 %) of primaries were left-sided in the present analysis and due to known differences between left- and right-sided colon cancers, data is neither comparable nor transferable.

Furthermore, we present survival data from our study population according to metastatic group. It is acknowledged that patients with CRC and lung metastasis live longer than those with other metastatic manifestations [31, 32]. Accordingly, in the present study, patients with lung metastasis showed longer OS and PFS than patients with liver metastasis. Furthermore, the known prognostic value of the MAPK pathway mutational status was clearly confirmed indicating that the patient cohort analyzed was representative of large CRC populations. Interestingly, no prognostic relevance of the CD133 and β-catenin expression status was observed thus seeming only predictive for the formation of metastasis but not decisive for long-term outcome.

Since most tumours of the investigated patient cohort derived from the left-sided colon and rectum, as expected, only few patients (2.9%) showed dMMR. Due to low numbers, statistical comparisons are not reasonable and the relevance of the MMR status stays unclear for the investigated questions of the present analysis. In the investigated study cohort, patients with primary tumours in the rectum were overrepresented in both metastatic groups (PUL and HEP) compared to the control group (M0). However, the in-depth analysis revealed no statistical difference between the PUL and the HEP group. Thus, results showing significant differences in single-biomarker alterations as well as biomarker combinations between these groups appear not to be biased by localization of the primary tumour. Furthermore, multivariate analysis ruled out confounding factors for PUL versus HEP patients.

Of interest, previous studies show that *KRAS* mutations are associated with metastasis to the central nervous system (CNS) [18, 23]. Thus, CRC patients with haematogenous spread to the lungs and to the CNS seem to have molecular features in common that possibly indicate similar carcinogenic pathways leading to metastasis formation in the lungs and/or brain. However, underlying mechanisms can certainly not be derived from the presented results.

The present analysis is certainly limited by its retrospective and exploratory design as well as data acquisition from a cancer registry. Thus, by its nature, the study cannot serve with results from functional studies or in vivo experiments but nevertheless provides interesting information about the presented biomarker alterations.

Taken together, our data indicate that the enhanced activity of the Wnt/β-catenin pathway intensified by a MAPK pathway mutation may play a crucial role in the formation of lung metastasis, whereas high expression of CD133 correlates with the presence of liver metastasis. Based on these findings, we raise the hypothesis that the anatomic site of metastasis formation may depend on different patterns or varying sequences of molecular marker alterations during CRC carcinogenesis, respectively. On this basis, we would like to initiate a discussion on the necessity of a novel and clinically relevant classification of CRC. Certainly, precise mechanisms underlying the metastasis formation in different anatomic still remain unclear. However, knowledge of the anticipated site of distant metastasis would substantially impact clinical management, so an increased effort into the identification of solid biomarkers for organotropic formation of metastasis is justified.

## Supplementary information

Online Resource 1: Consort diagram (DOCX 46 kb).

Online Resource 2: Antibodies used for the study (DOCX 18 kb).

Online Resource 3: Primer and PCR-Protocols used for the study (DOCX 23 kb).

Online Resource 4: Bar charts comparing frequencies of selected patient and tumour characteristics between groups. Abbreviations: PUL, patients with exclusive lung metastasis; HEP, patients with exclusive liver metastasis; M0, patients without metastatic disease (PNG 51 kb).

Online Resource 5: Results of the multivariate analysis. For calculation of the variable age the cohort was divided in the categories younger and older than 65 years (DOCX 20 kb).

Online Resource 6: Overall survival (OS) and progression-free survival (PFS) in the different patient cohorts with lung (PUL) and liver (HEP) metastasis. Survival data is presented as median months and ranges are depicted in brackets. Significant p-values are printed in bold. * Positive is defined as both markers being positive (positive for MAP kinase mutational status is defined as the presence of a mutation; positive for β-catenin and CD133 expression is defined as high expression). Negative is defined as one of the markers or both markers being negative (negative for MAP kinase mutational status is defined as wild-type; negative for β-catenin and CD133 expression is defined as low expression). (DOCX 24 kb).

Online Resource 7: Kaplan-Meier curves for OS and PFS comparing patients with exclusive lung (PUL) versus exclusive liver metastasis (HEP) (PNG 44 kb).

Online Resource 8: Kaplan-Meier curves showing OS depending on metastatic pattern and MAP kinase mutational status (PNG 192 kb).

## Data Availability

Data that support the findings of this study is available from the corresponding author (J.N.) upon reasonable request.
