# Supplementary material for: Biomarker alterations associated with distinct patterns of metastatic spread in colorectal cancer
Source: Virchows Arch. 2020 Dec 9;478(4):695–705. doi: 10.1007/s00428-020-02983-6 (PMC7990752; doi:10.1007/s00428-020-02983-6)
Supplement: Supplementary file 1 — Consort diagram (DOCX 46 kb). [file 428_2020_2983_MOESM1_ESM.docx]

**PUL group**

**HEP group**

**M0 (control) group**

**N=226**

Patients with CRC and exclusive lung metastasis

reported to the MCR through the

University Hospital of Munich or the

LMU Institute of Pathology

between 1994 and 2017

**N=73**

Patients with CRC

diagnosed before

1998 and after 2012

**N=153**

Patients with CRC and exclusive lung metastasis

reported to the MCR

between 1998 and 2012

**N=71**

Patients excluded due

to lacking tumor tissue

**N=82**

Patients with CRC and exclusive lung metastasis

and available tumor tissue

**N=1533**

Patients with CRC and exclusive liver metastasis

reported to the MCR through the

University Hospital of Munich or the

LMU Institute of Pathology

between 1989 and 2017

**N=377**

Patients with CRC

diagnosed before

1998 and after 2012

**N=1156**

Patients with CRC and exclusive liver metastasis reported to the MCR

between 1998 and 2012

**N=1085**

Patients not found suitable

for matched-pair analysis

**N=82**

Patients with CRC and exclusive liver metastasis and matching to PUL group

and available tumor tissue

**N=82**

Patients with CRC and exclusive liver metastasis matched to PUL group available tumor tissue

**N=2231**

Patients with non-metastatic CRC

reported to the MCR through the

University Hospital of Munich or the

Institute of Pathology

between 1989 and 2013

**N=1076**

Patients with CRC

diagnosed before

1998 and after 2012

**N=1155**

Patients with CRC and no documented distant metastases during a follow-up period of 5 years reported to the MCR between 1998 and 2012

**N=1084**

Patients not found suitable for matched-pair analysis

**N=82**

Patients with non-metastatic CRC

and matching to PUL group

and available tumor tissue

**N=82**

Patients with non-metastatic CRC matching to PUL group and available tumor tissue
