# Supplementary material for: Biomarker alterations associated with distinct patterns of metastatic spread in colorectal cancer
Source: Virchows Arch. 2020 Dec 9;478(4):695–705. doi: 10.1007/s00428-020-02983-6 (PMC7990752; doi:10.1007/s00428-020-02983-6)
Supplement: Supplementary file 2 — Antibodies used for the study (DOCX 18 kb). [file 428_2020_2983_MOESM2_ESM.docx]

**Online Resource 2: Antibodies used for the study**

| **Antibody** | **Company** | **Source** | **Clone** | **Dilution** | **Detection system** |
| --- | --- | --- | --- | --- | --- |
| **β-catenin** | Ventana Medical Systems, Oro Valley, AZ | mouse monoclonal | clone 14 | ready to use | Ventana OptiView DAB IHC Detection Kit |
| **p53** | Thermo Scientific, Kalamazoo, MI | mouse monoclonal | DO-7 | 1:1000 | Ventana OptiView DAB IHC Detection Kit |
| **CD133** | Miltenyi Biotec, Auburn, CA | mouse monoclonal | AC133 | 1:50 | Ventana OptiView DAB IHC Detection Kit |
| **MLH1** | Leica Biosystems, Newcastle upon Tyne, UK | mouse monoclonal | ES05 | 1:100 | Ventana UltraView DAB IHC Detection Kit |
| **MSH1** | Ventana Medical Systems, Oro Valley, AZ | mouse monoclonal | G219-1129 | ready to use | Ventana OptiView DAB IHC Detection Kit |
