# Supplementary material for: Biomarker alterations associated with distinct patterns of metastatic spread in colorectal cancer
Source: Virchows Arch. 2020 Dec 9;478(4):695–705. doi: 10.1007/s00428-020-02983-6 (PMC7990752; doi:10.1007/s00428-020-02983-6)
Supplement: Supplementary file 3 — Primer and PCR-Protocols used for the study (DOCX 23 kb). [file 428_2020_2983_MOESM3_ESM.docx]

**Online Resource 3: Primers used for the study**

| **Gene** | **Primer-Sequence** | **Tm** | **Number of Cycles** | **PCR Conditions** | **Polymerase** | **length PCR product [bp]** |
| --- | --- | --- | --- | --- | --- | --- |
| **BRAF Exon 15** | TGAAGACCTCACAGTAAAAATAGG | 60 | 50 | 30'' 94°C | Qiagen HotStar Taq | 91 |
|  | Biotin~TCCAGACAACTGTTCAAACTGAT |  |  | 30'' Tm |  |  |
| sequencing | GTAAAAATAGGTGATTTTGG |  |  | 30'' 72°C |  |  |
| sequence to analyse: | TC/GTAGCTACG/AG/AT/A/GG/TAAATCT |  |  |  |  |  |
| **KRAS Exon 2** | NNNGGCCTGCTGAAAATGACTGAA | 60 | 50 | 30'' 94°C | Qiagen HotStar Taq | 82 |
|  | Biotin~TTAGCTGTATCGTCAAGGCACTCT |  |  | 30'' Tm |  |  |
| sequencing | TGTGGTAGTTGGAGCT |  |  | 30'' 72°C |  |  |
| sequence to analyse: | GNTGRCGTAGGCAA |  |  |  |  |  |
| **KRAS Exon 3** | AATTGATGGAGAAACCTGTCTCTT | 60 | 50 | 30'' 95°C | Qiagen HotStar Taq | 78 |
|  | Biotin~TCCTCATGTACTGGTCCCTCATT |  |  | 30'' Tm |  |  |
| sequencing | TCTCTTGGATATTCTCGAC |  |  | 30'' 72°C |  |  |
| sequence to analyse: | ACAGCAGGTACGATCAGAGGAGTA |  |  |  |  |  |
| **KRAS Exon 4, codon 117** | CTGAAGATGTACCTATGGTCCTAG | 60 | 50 | 30'' 94°C | Qiagen HotStar Taq | 77 |
|  | Biotin~CTGAGCCTGTTTTGTGTCTACTG |  |  | 30'' Tm |  |  |
| sequencing | ACCTATGGTCCTAGTAGGAA |  |  | 30'' 72°C |  |  |
| sequence to analyse: | ATAAA/C/TTGTGATTTGCCTTC |  |  |  |  |  |
| **KRAS Exon 4, codon 146** | GGCTCAGGACTTAGCAAGAAGTTA | 60 | 50 | 30'' 95°C | Qiagen HotStar Taq | 114 |
|  | Biotin~AGTTATGATTTTGCAGAAAACAGA |  |  | 30'' Tm |  |  |
| sequencing | GAATTCCTTTTATTGAAAC |  |  | 30'' 72°C |  |  |
| sequence to analyse: | ATCAG/A/CC/TAAAGACAA |  |  |  |  |  |
| **NRAS Exon 2** | CTTGCTGGTGTGAAATGACTGAG | 60 | 50 | 30'' 95°C | Qiagen HotStar Taq | 79 |
|  | Biotin~TGGATTGTCAGTGCGCTTTT |  |  | 30'' Tm |  |  |
| sequencing | TGGTGGTGGTTGGAG |  |  | 30'' 72°C |  |  |
| sequence to analyse: | CAG/A/C/TGTG/CG/ATGTT |  |  |  |  |  |
| **NRAS Exon 3** | AAACCTGTTTGTTGGACATACTG | 60 | 50 | 30'' 94°C | Qiagen HotStar Taq | 67 |
|  | Biotin~TATTGGTCTCTCATGGCACTGT |  |  | 30'' Tm |  |  |
| sequencing | TTGTTGGACATACTGGAT |  |  | 30'' 72°C |  |  |
| sequence to analyse: | ACAGCTGGACA/T/GAG/C/TAGA |  |  |  |  |  |
| **NRAS Exon 4, codon 117** | Biotin~ATGATGTACCTATGGTGCTAGTGG | 60 | 50 | 30'' 94°C | Qiagen HotStar Taq | 93 |
|  | CGTAACTCTTGGCCAGTTCG |  |  | 30'' Tm |  |  |
| sequencing | TCCTTGTTGGCAAATC |  |  | 30'' 72°C |  |  |
| sequence to analyse: | ACACNTGTTTCCCACTAG |  |  |  |  |  |
| **NRAS Exon 4, codon 146** | GCAACTGGCCAAGAGTTACG | 60 | 50 | 30'' 94°C | Qiagen HotStar Taq | 75 |
|  | Biotin~TGAAAGCTGTACCATACCTGTCTG |  |  | 30'' Tm |  |  |
| sequencing | TCCATTCATTGAAACCT |  |  | 30'' 72°C |  |  |
| sequence to analyse: | TCARCCAAGACCAG |  |  |  |  |  |
