# Supplementary figures and images for: Biomarker alterations associated with distinct patterns of metastatic spread in colorectal cancer
Source: Virchows Arch. 2020 Dec 9;478(4):695–705. doi: 10.1007/s00428-020-02983-6 (PMC7990752; doi:10.1007/s00428-020-02983-6)

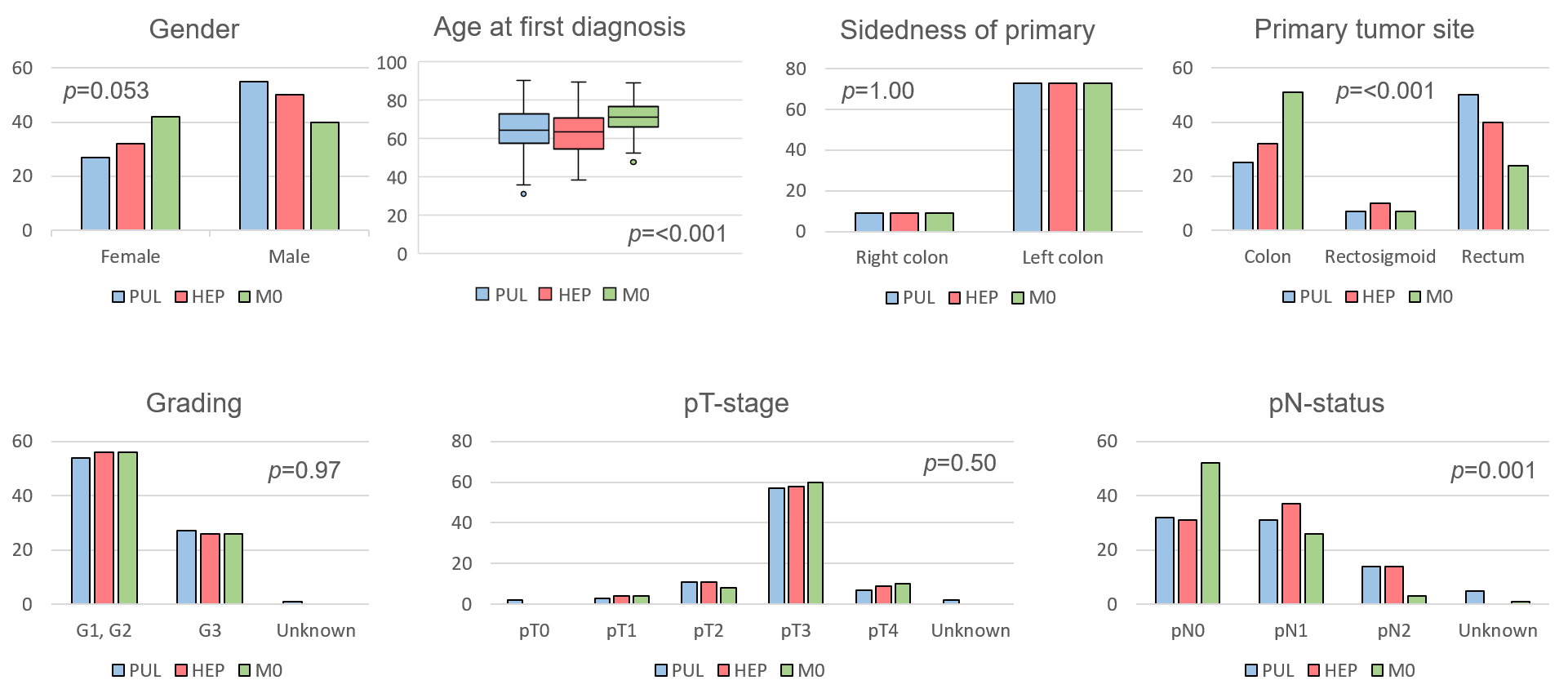

Supplement: Supplementary file 4 — Bar charts comparing frequencies of selected patient and tumour characteristics between groups. Abbreviations: PUL, patients with exclusive lung metastasis; HEP, patients with exclusive liver metastasis; M0, patients without metastatic disease (PNG 51 kb). [file 428_2020_2983_MOESM4_ESM.png]

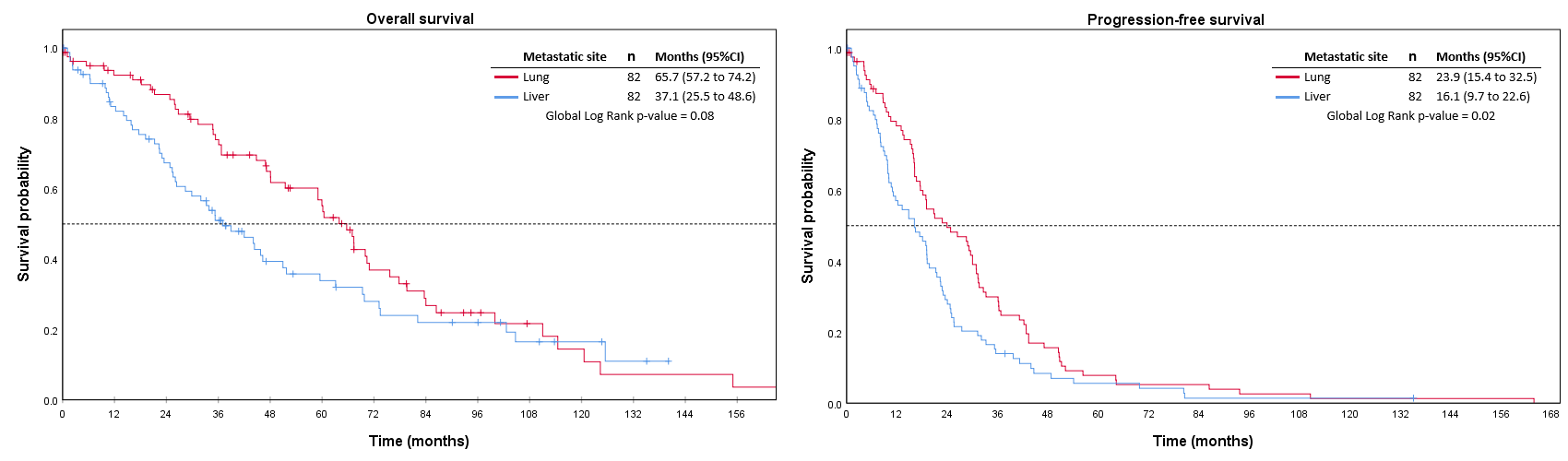

Supplement: Supplementary file 7 — Kaplan-Meier curves for OS and PFS comparing patients with exclusive lung (PUL) versus exclusive liver metastasis (HEP) (PNG 44 kb). [file 428_2020_2983_MOESM7_ESM.png]

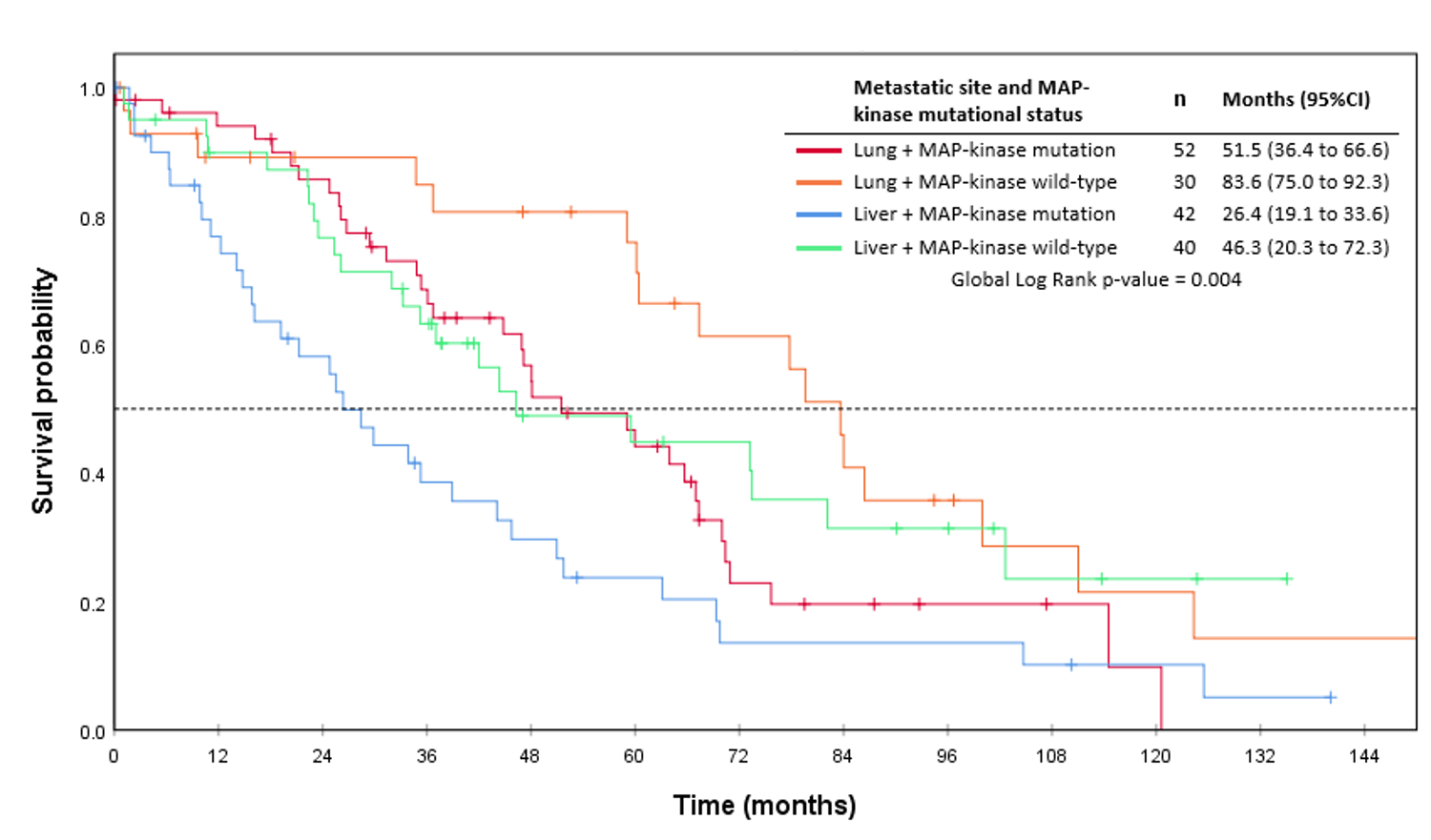

Supplement: Supplementary file 8 — Kaplan-Meier curves showing OS depending on metastatic pattern and MAP kinase mutational status (PNG 192 kb). [file 428_2020_2983_MOESM8_ESM.png]
