# Supplementary material for: Biomarker alterations associated with distinct patterns of metastatic spread in colorectal cancer
Source: Virchows Arch. 2020 Dec 9;478(4):695–705. doi: 10.1007/s00428-020-02983-6 (PMC7990752; doi:10.1007/s00428-020-02983-6)
Supplement: Supplementary file 5 — Results of the multivariate analysis. For calculation of the variable age the cohort was divided in the categories younger and older than 65 years (DOCX 20 kb). [file 428_2020_2983_MOESM5_ESM.docx]

**Online Resource 5: Results of the multivariate analysis**

|  | **M0 🡪 M1** | **M0 🡪 PUL** | **M0 🡪 HEP** | **PUL 🡪 HEP** |
| --- | --- | --- | --- | --- |
| **Biomarker** | ***P***  **Odds Ratio (95%CI)** | ***P***  **Odds Ratio (95%CI)** | ***P***  **Odds Ratio (95%CI)** | ***P***  **Odds Ratio (95%CI)** |
| Age at first diagnosis | **<0.001**  0.26 [0.14; 0.50] | **<0.001**  0.26 [0.13; 0.55] | **<0.001**  0.25 [0.12; 0.51] | 0.93  0.97 [0.51; 1.86] |
| Nodal-status | **0.01**  2.33 [1.27; 4.27] | **0.04**  2.17 [1.05; 4.50] | **0.01**  2.66 [1.30; 5.44] | 0.92  1.03 [0.53; 2.01] |
| MAP-kinase mutational status | 0.26  1.42 [0.77; 2.60] | **0.046**  2.07 [1.01; 4.24] | 0.94  0.97 [0.47; 2.01] | 0.09  0.55 [0.27; 1.09] |
| MMR-status | 0.16  0.28 [0.05; 1.68] | 0.25  0.23 [0.02; 2.84] | 0.18  0.22 [0.03; 1.97] | 0.74  1.55 [0.12; 19.88] |
| p53 status | 0.91  1.04 [0.54; 1.99] | 0.96  1.02 [0.48; 2.18] | 0.88  1.06 [0.49; 2.31] | 0.48  1.29 [0.64; 2.62] |
| β-catenin expression | 0.53  1.22 [0.66; 2.25] | 0.15  1.70 [0.82; 3.51] | 0.78  0.90 [0.43; 1.87] | 0.12  0.59 [0.30; 1.16] |
| CD133 expression | 0.054  2.12 [0.99; 4.54] | 0.23  1.74 [0.71; 4.26] | **0.02**  2.94 [1.22; 7.09] | 0.30  1.48 [0.71; 3.07] |
