# Supplementary material for: Biomarker alterations associated with distinct patterns of metastatic spread in colorectal cancer
Source: Virchows Arch. 2020 Dec 9;478(4):695–705. doi: 10.1007/s00428-020-02983-6 (PMC7990752; doi:10.1007/s00428-020-02983-6)
Supplement: Supplementary file 6 — Overall survival (OS) and progression-free survival (PFS) in the different patient cohorts with lung (PUL) and liver (HEP) metastasis. Survival data is presented as median months and ranges are depicted in brackets. Significant p-values are printed in bold. * Positive is defined as both markers being positive (positive for MAP kinase mutational status is defined as the presence of a mutation; positive for β-catenin and CD133 expression is defined as high expression). Negative is defined as one of the markers or both markers being negative (negative for MAP kinase mutational status is defined as wild-type; negative for β-catenin and CD133 expression is defined as low expression). (DOCX 24 kb). [file 428_2020_2983_MOESM6_ESM.docx]

**Online Resource 5: Overall survival (OS) and progression-free survival (PFS) in the different patient cohorts with lung (PUL) and liver (HEP)** **metastasis**

|  | **Total**  **N=164** | **PUL**  **N=82** | | | **HEP**  **N=82** | | | **Global *P***  **Hazard Ratio** |
| --- | --- | --- | --- | --- | --- | --- | --- | --- |
| OS | N (PUL) = 53/82  N (HEP) = 56/82 | 65.7 [57.2; 74.2] | | | 37.1 [25.5; 48.6] | | | 0.08  1.41 [0.96; 2.06] |
| PFS | N (PUL) = 77/82  N (HEP) = 77/82 | 23.9 [15.4; 32.5] | | | 16.1 [9.7; 22.6] | | | **0.02**  1.45 [1.06; 2.00] |
| MAP-kinase mutational status | | mutation | wild-type | *P*  Hazard Ratio | mutation | wild-type | *P*  Hazard Ratio | Global *P* |
| OS | | 51.5 [36.4; 66.6] | 83.6 [75.0; 92.3] | **0.03**  1.96 [1.07; 3.60] | 26.4 [19.1; 33.6] | 46.3 [20.3; 72.3] | **0.02**  1.93 [1.13; 3.29] | **0.004** |
| PFS | | 19.0 [11.5; 26.6] | 28.8 [15.1; 42.6] | **0.01**  1.99 [1.18; 3.35] | 10.1 [5.8; 14.3] | 19.1 [15.5; 22.6] | 0.07  1.53 [0.97; 2.42] | **0.002** |
| CD133 expression | | high | low | *P*  Hazard Ratio | high | low | *P*  Hazard Ratio | Global *P* |
| OS | | 59.0 [38.7; 79.4] | 67.4 [55.9; 78.9] | 0.25  1.42 [0.78; 2.61] | 63.1 [30.3; 96.0] | 33.9 [23.7; 44.1] | 0.16  0.66 [0.37; 1.19] | 0.08 |
| PFS | | 22.8 [15.0; 30.5] | 26.4 [13.7; 39.1] | 0.20  1.42 [0.83; 2.43] | 14.8 [4.5; 25.1] | 19.1 [11.9; 26.2] | 0.78  0.93 [0.57; 1.53] | 0.07 |
| β-catenin expression | | high | low | *P*  Hazard Ratio | high | low | *P*  Hazard Ratio | Global *P* |
| OS | | 67.4 [54.8; 80.0] | 60.0 [39.8; 80.1] | 0.38  0.79 [0.46; 1.35] | 33.9 [14.5; 53.2] | 42.0 [29.4; 54.5] | 0.70  0.90 [0.52; 1.56] | 0.28 |
| PFS | | 24.8 [13.7; 35.9] | 23.9 [10.5; 37.4] | 0.83  0.95 [0.60; 1.52] | 19.1 [14.4; 23.8] | 12.2 [7.0; 17.5] | 0.24  0.76 [0.48; 1.20] | 0.07 |
| MAP-kinase mutational status  **PLUS** β-catenin expression* | | positive | negative | *P*  Hazard Ratio | positive | negative | *P*  Hazard Ratio | Global *P* |
| OS | | 67.0 [39.4; 94.5] | 63.9 [55.6; 72.3] | 0.91  0.97 [0.51; 1.82] | 33.9 [14.8; 52.9] | 37.1 [25.0; 49.1] | 0.61  1.21 [0.57; 2.58] | 0.31 |
| PFS | | 22.8 [14.0; 31.5] | 26.4 [14.0; 38.8] | 0.07  1.63 [0.96; 2.79] | 19.1 [11.2; 27.0] | 14.8 [8.3; 21.3] | 0.83  0.93 [0.48; 1.82] | 0.07 |
| MAP-kinase mutational status  **PLUS** CD133 expression* | | positive | negative | *P*  Hazard Ratio | positive | negative | *P*  Hazard Ratio | Global *P* |
| OS | | 48.1 [27.7; 68.5] | 67.4 [55.6; 79.2] | **0.01**  2.37 [1.20; 4.67] | 44.1 [24.6; 63.5] | 35.3 [23.7; 46.9] | 0.54  1.22 [0.65; 2.28] | 0.05 |
| PFS | | 19.0 [16.3; 21.8] | 28.5 [18.8; 38.2] | **0.04**  1.86 [1.01; 3.43] | 9.8 [8.1; 11.6] | 18.1 [13.0; 23.2] | 0.24  1.38 [0.80; 2.38] | **0.03** |
| β-catenin expression  **PLUS** CD133 expression* | | positive | negative | *P*  Hazard Ratio | positive | negative | *P*  Hazard Ratio | Global *P* |
| OS | | 60.4 [56.9; 63.9] | 65.7 [56.8; 74.6] | 0.59  1.27 [0.53; 3.02] | 45.8 [29.2; 62.3] | 35.3 [22.6; 48.0] | 0.21  0.56 [0.22; 1.40] | 0.16 |
| PFS | | 22.8 [14.8; 30.8] | 23.9 [12.4; 35.5] | 0.26  1.53 [0.72; 3.27] | 16.4 [13.3; 19.5] | 14.8 [7.3; 22.3] | 0.44  0.76 [0.38; 1.53] | 0.06 |
